# (*E*)-5-Benzyl-1-methyl-*N*-nitro-1,3,5-triazinan-2-imine

**DOI:** 10.1107/S1600536810009426

**Published:** 2010-03-20

**Authors:** Liang-Zhong Xu, Rui-Feng Yin, Hong-Xin Li

**Affiliations:** a College of Chemistry and Molecular Engineering, Qingdao University of Science and Technology, Qingdao 266042, People’s Republic of China

## Abstract

In the title compound, C_11_H_15_N_5_O_2_, the 1,3,5-triazine ring exhibits a half-chair conformation. An intra­molecular N—H⋯O inter­action occurs. In the crystal structure, mol­ecules are connected by inter­molecular C—H⋯O and N—H⋯N hydrogen bonds, forming a zigzag chain along the *b* axis.

## Related literature

For the synthesis of the title compound, see: Ebihara *et al.* (1998 ▶). For related structures, see: Hu *et al.* (2008 ▶); Zhao *et al.* (2008 ▶).
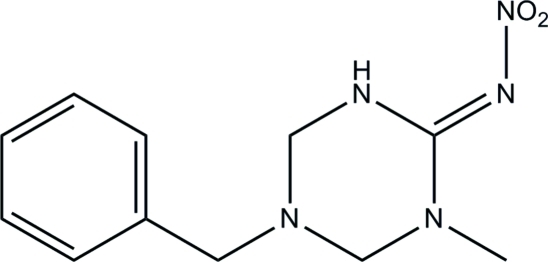


## Experimental

### 

#### Crystal data

C_11_H_15_N_5_O_2_
*M* *_r_* = 249.28
Monoclinic, 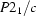

*a* = 12.293 (3) Å
*b* = 6.7769 (14) Å
*c* = 14.858 (3) Å
β = 107.36 (3)°
*V* = 1181.5 (4) Å^3^
*Z* = 4
Mo *K*α radiation
μ = 0.10 mm^−1^
*T* = 293 K
0.45 × 0.13 × 0.10 mm

#### Data collection

Rigaku R-AXIS RAPID IP area-detector diffractometer
Absorption correction: multi-scan (*ABSCOR*; Higashi, 1995 ▶) *T*_min_ = 0.956, *T*_max_ = 0.990
10812 measured reflections
2697 independent reflections
2215 reflections with *I* > 2σ(*I*)
*R*_int_ = 0.026

#### Refinement

*R*[*F* ^2^ > 2σ(*F* ^2^)] = 0.043
*wR*(*F* ^2^) = 0.141
*S* = 1.15
2697 reflections
164 parameters
H-atom parameters constrained
Δρ_max_ = 0.33 e Å^−3^
Δρ_min_ = −0.28 e Å^−3^

### 

Data collection: *RAPID-AUTO* (Rigaku, 2004 ▶); cell refinement: *RAPID-AUTO*; data reduction: *RAPID-AUTO*; program(s) used to solve structure: *SHELXS97* (Sheldrick, 2008 ▶); program(s) used to refine structure: *SHELXL97* (Sheldrick, 2008 ▶); molecular graphics: *SHELXTL* (Sheldrick, 2008 ▶); software used to prepare material for publication: *SHELXTL*.

## Supplementary Material

Crystal structure: contains datablocks I, global. DOI: 10.1107/S1600536810009426/is2529sup1.cif

Structure factors: contains datablocks I. DOI: 10.1107/S1600536810009426/is2529Isup2.hkl

Additional supplementary materials:  crystallographic information; 3D view; checkCIF report

## Figures and Tables

**Table 1:** Hydrogen-bond geometry (Å, °)

*D*—H⋯*A*	*D*—H	H⋯*A*	*D*⋯*A*	*D*—H⋯*A*
N1—H1*A*⋯N4^i^	0.86	2.27	3.093 (2)	162
C3—H3*A*⋯O2^ii^	0.97	2.59	3.305 (2)	131
N1—H1*A*⋯O1	0.86	2.33	2.730 (2)	109

Symmetry codes: (i) 
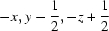
; (ii) 
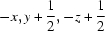
.

**Fig. 1.**
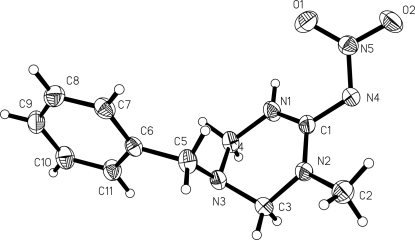
View of the title compound (I), with displacement ellipsoids drawn at the 40% probability level.

## supplementary crystallographic information

### Comment

The title compound was synthesized as an intermediate for the synthesis of clothianidin (Ebihara *et al.*, 1998). We report here the crystal structure of the title compound, (I).

In (I) (Fig. 1), all bond lengths and angles are normal and in a good agreement with those reported previously (Hu *et al.*, 2008). The 1,3,5-triazine ring (C1/C3/C4/N1—N3) exhibits a half-chair conformation. The crystal structure is stabilized by intermolecular C–H···O and N–H···N hydrogen bonds.

### Experimental

1-Methyl-2-nitroguanidine 1.18 g (10 mmol) and 2.5 g formaldehyde (concentration 36%, 30 mmol) was dissolved in 20 ml ethanol, then phenylmethanamine (10 mmol) was added dropwise during 30 min at 30-40 °C. After this addition, the reaction mixture was heated with stirring for three hours at 30-40 °C. The mixture was cooled to room temperature and filtered to afford title compound 2.39 g (yield 96%). Single crystals suitable for X-ray diffraction were obtained by recrystallization from ethanol at room temperature.

### Refinement

All H atoms were placed in calculated positions, with C–H = 0.93-0.97 Å and N–H = 0.86 Å, and included in the final cycles of refinement using a riding model, with *U*_iso_(H) = 1.2*U*_eq_(C, N) for aryl, methylene and N-bounded H atoms and 1.5*U*_eq_(C) for methyl H atoms.

### Crystal data

**Table:**

C_11_H_15_N_5_O_2_	*F*(000) = 528
*M**_r_* = 249.28	*D*_x_ = 1.401 Mg m^−^^3^
Monoclinic, *P*2_1_/*c*	Mo *K*α radiation, λ = 0.71073 Å
Hall symbol: -P 2ybc	Cell parameters from 2501 reflections
*a* = 12.293 (3) Å	θ = 2.3–25.1°
*b* = 6.7769 (14) Å	µ = 0.10 mm^−^^1^
*c* = 14.858 (3) Å	*T* = 293 K
β = 107.36 (3)°	Needle, colorless
*V* = 1181.5 (4) Å^3^	0.45 × 0.13 × 0.10 mm
*Z* = 4	

### Data collection

**Table:**

Rigaku R-AXIS RAPID IP area-detector diffractometer	2697 independent reflections
Radiation source: Rotating Anode	2215 reflections with *I* > 2σ(*I*)
graphite	*R*_int_ = 0.026
ω scans	θ_max_ = 27.5°, θ_min_ = 3.3°
Absorption correction: multi-scan (*ABSCOR*; Higashi, 1995)	*h* = −15→15
*T*_min_ = 0.956, *T*_max_ = 0.990	*k* = −8→8
10812 measured reflections	*l* = −19→19

### Refinement

**Table:**

Refinement on *F*^2^	Secondary atom site location: difference Fourier map
Least-squares matrix: full	Hydrogen site location: inferred from neighbouring sites
*R*[*F*^2^ > 2σ(*F*^2^)] = 0.043	H-atom parameters constrained
*wR*(*F*^2^) = 0.141	*w* = 1/[σ^2^(*F*_o_^2^) + (0.0641*P*)^2^ + 0.4408*P*] where *P* = (*F*_o_^2^ + 2*F*_c_^2^)/3
*S* = 1.15	(Δ/σ)_max_ < 0.001
2697 reflections	Δρ_max_ = 0.33 e Å^−^^3^
164 parameters	Δρ_min_ = −0.28 e Å^−^^3^
0 restraints	Extinction correction: *SHELXL97* (Sheldrick, 2008), Fc^*^=kFc[1+0.001xFc^2^λ^3^/sin(2θ)]^-1/4^
Primary atom site location: structure-invariant direct methods	Extinction coefficient: 0.053 (5)

### Special details

**Table:**

Geometry. All esds (except the esd in the dihedral angle between two l.s. planes) are estimated using the full covariance matrix. The cell esds are taken into account individually in the estimation of esds in distances, angles and torsion angles; correlations between esds in cell parameters are only used when they are defined by crystal symmetry. An approximate (isotropic) treatment of cell esds is used for estimating esds involving l.s. planes.
Refinement. Refinement of *F*^2^ against ALL reflections. The weighted *R*-factor wR and goodness of fit *S* are based on *F*^2^, conventional *R*-factors *R* are based on *F*, with *F* set to zero for negative *F*^2^. The threshold expression of *F*^2^ > σ(*F*^2^) is used only for calculating *R*-factors(gt) etc. and is not relevant to the choice of reflections for refinement. *R*-factors based on *F*^2^ are statistically about twice as large as those based on *F*, and *R*- factors based on ALL data will be even larger.

### Fractional atomic coordinates and isotropic or equivalent isotropic displacement parameters (Å^2^)

**Table:**

	*x*	*y*	*z*	*U*_iso_*/*U*_eq_	
O1	−0.08476 (12)	0.2664 (2)	0.32912 (13)	0.0648 (5)	
O2	−0.20472 (10)	0.4688 (2)	0.23990 (9)	0.0491 (4)	
N1	0.13187 (12)	0.3877 (2)	0.34487 (10)	0.0396 (4)	
H1A	0.0946	0.2966	0.3081	0.048*	
N2	0.13204 (11)	0.69132 (19)	0.41305 (9)	0.0337 (3)	
N3	0.29652 (11)	0.4822 (2)	0.47169 (9)	0.0324 (3)	
N4	−0.03718 (11)	0.5836 (2)	0.31457 (11)	0.0399 (4)	
N5	−0.10875 (11)	0.4321 (2)	0.29433 (9)	0.0342 (3)	
C1	0.07548 (13)	0.5462 (2)	0.35840 (10)	0.0320 (3)	
C2	0.07568 (15)	0.8650 (3)	0.43518 (14)	0.0446 (4)	
H2A	−0.0053	0.8513	0.4084	0.067*	
H2B	0.1011	0.9799	0.4094	0.067*	
H2C	0.0940	0.8786	0.5024	0.067*	
C3	0.25803 (13)	0.6801 (2)	0.44754 (12)	0.0379 (4)	
H3A	0.2893	0.7288	0.3991	0.046*	
H3B	0.2858	0.7639	0.5025	0.046*	
C4	0.25463 (13)	0.3596 (3)	0.38930 (11)	0.0354 (4)	
H4B	0.2694	0.2222	0.4071	0.043*	
H4C	0.2951	0.3913	0.3443	0.043*	
C5	0.26964 (14)	0.4064 (3)	0.55497 (11)	0.0370 (4)	
H5A	0.1889	0.3765	0.5381	0.044*	
H5B	0.2855	0.5083	0.6030	0.044*	
C6	0.33636 (12)	0.2241 (2)	0.59520 (10)	0.0305 (3)	
C7	0.29232 (14)	0.0894 (3)	0.64553 (11)	0.0366 (4)	
H7A	0.2200	0.1099	0.6516	0.044*	
C8	0.35422 (16)	−0.0750 (3)	0.68681 (12)	0.0420 (4)	
H8A	0.3239	−0.1632	0.7209	0.050*	
C9	0.46112 (16)	−0.1076 (3)	0.67726 (12)	0.0426 (4)	
H9A	0.5029	−0.2181	0.7046	0.051*	
C10	0.50547 (14)	0.0239 (3)	0.62723 (12)	0.0426 (4)	
H10A	0.5775	0.0018	0.6209	0.051*	
C11	0.44426 (13)	0.1892 (3)	0.58611 (11)	0.0376 (4)	
H11A	0.4753	0.2770	0.5524	0.045*	

### Atomic displacement parameters (Å^2^)

**Table:**

	*U*^11^	*U*^22^	*U*^33^	*U*^12^	*U*^13^	*U*^23^
O1	0.0479 (8)	0.0396 (8)	0.1065 (13)	−0.0004 (6)	0.0227 (8)	0.0232 (8)
O2	0.0311 (6)	0.0622 (9)	0.0465 (7)	−0.0060 (6)	0.0002 (5)	−0.0005 (6)
N1	0.0354 (7)	0.0328 (7)	0.0410 (8)	0.0066 (6)	−0.0033 (6)	−0.0094 (6)
N2	0.0310 (7)	0.0272 (7)	0.0375 (7)	0.0028 (5)	0.0022 (5)	−0.0029 (5)
N3	0.0301 (6)	0.0355 (7)	0.0283 (6)	0.0030 (5)	0.0038 (5)	0.0022 (5)
N4	0.0302 (7)	0.0320 (7)	0.0485 (8)	−0.0010 (6)	−0.0020 (6)	0.0041 (6)
N5	0.0324 (7)	0.0367 (7)	0.0341 (7)	−0.0014 (6)	0.0106 (5)	0.0000 (5)
C1	0.0325 (7)	0.0290 (7)	0.0297 (7)	0.0025 (6)	0.0019 (6)	0.0016 (6)
C2	0.0442 (9)	0.0344 (9)	0.0510 (10)	0.0073 (7)	0.0079 (8)	−0.0097 (7)
C3	0.0306 (8)	0.0341 (8)	0.0423 (9)	−0.0016 (6)	0.0005 (6)	0.0018 (7)
C4	0.0336 (8)	0.0413 (9)	0.0296 (7)	0.0088 (7)	0.0065 (6)	−0.0003 (6)
C5	0.0372 (8)	0.0441 (9)	0.0301 (8)	0.0096 (7)	0.0105 (6)	0.0018 (7)
C6	0.0297 (7)	0.0363 (8)	0.0231 (6)	0.0011 (6)	0.0042 (5)	−0.0020 (6)
C7	0.0329 (7)	0.0467 (9)	0.0296 (7)	−0.0024 (7)	0.0086 (6)	−0.0020 (7)
C8	0.0502 (10)	0.0419 (9)	0.0324 (8)	−0.0064 (8)	0.0101 (7)	0.0030 (7)
C9	0.0486 (10)	0.0378 (9)	0.0346 (8)	0.0066 (8)	0.0021 (7)	0.0045 (7)
C10	0.0339 (8)	0.0483 (10)	0.0442 (9)	0.0103 (7)	0.0094 (7)	0.0042 (8)
C11	0.0326 (8)	0.0449 (9)	0.0357 (8)	0.0022 (7)	0.0109 (6)	0.0071 (7)

### Geometric parameters (Å, °)

**Table:**

O1—N5	1.2350 (19)	C3—H3B	0.9700
O2—N5	1.2404 (18)	C4—H4B	0.9700
N1—C1	1.326 (2)	C4—H4C	0.9700
N1—C4	1.468 (2)	C5—C6	1.505 (2)
N1—H1A	0.8600	C5—H5A	0.9700
N2—C1	1.332 (2)	C5—H5B	0.9700
N2—C2	1.452 (2)	C6—C7	1.388 (2)
N2—C3	1.4811 (19)	C6—C11	1.393 (2)
N3—C3	1.431 (2)	C7—C8	1.385 (2)
N3—C4	1.442 (2)	C7—H7A	0.9300
N3—C5	1.466 (2)	C8—C9	1.381 (3)
N4—N5	1.3269 (19)	C8—H8A	0.9300
N4—C1	1.367 (2)	C9—C10	1.373 (3)
C2—H2A	0.9600	C9—H9A	0.9300
C2—H2B	0.9600	C10—C11	1.385 (2)
C2—H2C	0.9600	C10—H10A	0.9300
C3—H3A	0.9700	C11—H11A	0.9300
			
C1—N1—C4	123.43 (14)	N3—C4—H4B	109.3
C1—N1—H1A	118.3	N1—C4—H4B	109.3
C4—N1—H1A	118.3	N3—C4—H4C	109.3
C1—N2—C2	122.62 (13)	N1—C4—H4C	109.3
C1—N2—C3	118.34 (13)	H4B—C4—H4C	108.0
C2—N2—C3	118.93 (13)	N3—C5—C6	112.94 (12)
C3—N3—C4	108.64 (12)	N3—C5—H5A	109.0
C3—N3—C5	113.51 (13)	C6—C5—H5A	109.0
C4—N3—C5	113.66 (14)	N3—C5—H5B	109.0
N5—N4—C1	118.25 (13)	C6—C5—H5B	109.0
O1—N5—O2	121.13 (14)	H5A—C5—H5B	107.8
O1—N5—N4	123.34 (14)	C7—C6—C11	118.43 (15)
O2—N5—N4	115.48 (14)	C7—C6—C5	120.00 (14)
N1—C1—N2	119.30 (14)	C11—C6—C5	121.53 (14)
N1—C1—N4	125.42 (14)	C8—C7—C6	121.10 (15)
N2—C1—N4	115.07 (14)	C8—C7—H7A	119.5
N2—C2—H2A	109.5	C6—C7—H7A	119.5
N2—C2—H2B	109.5	C9—C8—C7	119.81 (16)
H2A—C2—H2B	109.5	C9—C8—H8A	120.1
N2—C2—H2C	109.5	C7—C8—H8A	120.1
H2A—C2—H2C	109.5	C10—C9—C8	119.68 (16)
H2B—C2—H2C	109.5	C10—C9—H9A	120.2
N3—C3—N2	111.61 (13)	C8—C9—H9A	120.2
N3—C3—H3A	109.3	C9—C10—C11	120.81 (16)
N2—C3—H3A	109.3	C9—C10—H10A	119.6
N3—C3—H3B	109.3	C11—C10—H10A	119.6
N2—C3—H3B	109.3	C10—C11—C6	120.15 (15)
H3A—C3—H3B	108.0	C10—C11—H11A	119.9
N3—C4—N1	111.46 (13)	C6—C11—H11A	119.9
			
C1—N4—N5—O1	15.3 (2)	C5—N3—C4—N1	77.68 (17)
C1—N4—N5—O2	−167.27 (14)	C1—N1—C4—N3	20.6 (2)
C4—N1—C1—N2	1.7 (2)	C3—N3—C5—C6	−164.43 (13)
C4—N1—C1—N4	176.15 (15)	C4—N3—C5—C6	70.73 (17)
C2—N2—C1—N1	−176.81 (16)	N3—C5—C6—C7	−153.33 (14)
C3—N2—C1—N1	7.1 (2)	N3—C5—C6—C11	28.9 (2)
C2—N2—C1—N4	8.2 (2)	C11—C6—C7—C8	0.7 (2)
C3—N2—C1—N4	−167.92 (14)	C5—C6—C7—C8	−177.09 (14)
N5—N4—C1—N1	34.1 (2)	C6—C7—C8—C9	−0.7 (3)
N5—N4—C1—N2	−151.22 (15)	C7—C8—C9—C10	0.3 (3)
C4—N3—C3—N2	59.01 (17)	C8—C9—C10—C11	0.0 (3)
C5—N3—C3—N2	−68.49 (17)	C9—C10—C11—C6	0.1 (3)
C1—N2—C3—N3	−38.5 (2)	C7—C6—C11—C10	−0.4 (2)
C2—N2—C3—N3	145.25 (15)	C5—C6—C11—C10	177.37 (15)
C3—N3—C4—N1	−49.73 (18)		

### Hydrogen-bond geometry (Å, °)

**Table:**

*D*—H···*A*	*D*—H	H···*A*	*D*···*A*	*D*—H···*A*
N1—H1A···N4^i^	0.86	2.27	3.093 (2)	162
C3—H3A···O2^ii^	0.97	2.59	3.305 (2)	131
N1—H1A···O1	0.86	2.33	2.730 (2)	109

Symmetry codes: (i) −*x*, *y*−1/2, −*z*+1/2; (ii) −*x*, *y*+1/2, −*z*+1/2.
